# Predicting wildfires in Algerian forests using machine learning models

**DOI:** 10.1016/j.heliyon.2023.e18064

**Published:** 2023-07-10

**Authors:** Abdelhamid Zaidi

**Affiliations:** Department of Mathematics, College of Science, Qassim University, P.O.Box 6644, Buraydah 51452, KSA Saudi Arabia

**Keywords:** Wildfire forecasting, Artificial intelligence, Machine learning, Principal component analysis, Cross-validation

## Abstract

Algeria is one of the Maghreb countries most affected by wildfires. The economic, environmental, and societal consequences of these fires can last several years after the wildfire. Often, it is possible to avoid such disasters if the detection of the outbreak of fire is fast enough, reliable, and early. The lack of datasets has limited the methods used to predict wildfires in Algeria to the mapping risk areas, which is updated annually. This study is the result of the availability of a recent dataset relating the history of forest fires in the cities of Bejaia and Sidi Bel-Abbes during the year 2012. The dataset being small size, we used principal component analysis to reduce the number of variables to 6, while retaining 96.65% of the total variance. Moreover, we developed an artificial neural network (ANN) with two hidden layers to predict wildfires in these cities. Next, we trained and compared the performance of our classifier with those provided by the Logistic Regression, *K* Nearest Neighbors, Support Vector Machine, and Random Forest classifiers, using a 10-fold stratified cross-validation. The experiment shows a slight superiority of the ANN classifier compared to the others, in terms of accuracy, precision, and recall. Our classifier achieves an accuracy of 0.967±0.026 and F1-score of 0.971±0.023. The SHAP technique revealed the importance of the features (RH, DC, ISI) in the predictions of the ANN model.

## 1 Introduction

Forest fires are uncontrolled fires that occur in forests or other types of vegetation. They can be caused by natural factors such as lightning, volcanic eruptions, or spontaneous combustion, or by human factors such as arson, negligence, or land clearing. They can be influenced by various factors such as weather, vegetation, topography, and human activities. Forest fires can have devastating impacts on the environment, biodiversity, and human lives. They can destroy habitats, release greenhouse gases, reduce air quality, damage infrastructure, and endanger health and safety.

Predicting forest fires is a crucial task for preventing and managing these disasters. Forest fire prediction aims to estimate the likelihood, location, size, spread, intensity, and duration of a fire event based on various factors and data sources. Forest fire prediction can help decision-makers to allocate resources, plan mitigation strategies, deploy firefighting crews, issue warnings, and evaluate risks. However, forest fire prediction is a complex and challenging task that involves uncertainty, variability, nonlinearity, and high dimensionality of the data and the phenomena.

### 1.1 Overview of fire prediction methods

In this section, we review forest fire forecasting approaches that we have encountered in the literature. Based on our readings, we have identified three main approaches in which the majority of forest fire forecasting methods fall, physics-based models, statistical models and machine learning models. Here we give a brief description of these approaches, compare their advantages and disadvantages, and discuss their applications and limitations.

1) *Physics-based models*: These models use physical laws and equations to simulate the fire behavior and spread based on fuel characteristics, wind speed and direction, slope, moisture content, etc. They can provide accurate and detailed information on fire dynamics, but they require a lot of input data and computational resources, and they may not account for all the uncertainties and complexities involved in real fire scenarios. An example of a physics-based model is the Fire Dynamics Simulator (FDS), which is a computational fluid dynamics model that simulates fire-driven fluid flow and heat transfer. It can model fire spread and behavior in complex geometries and scenarios [1].
2) *Statistical models*: These models use historical data and statistical techniques to establish empirical relationships between fire occurrence and explanatory variables such as weather, vegetation, human activities, etc. Statistical models can provide simple and fast predictions based on available data, but they may not capture the nonlinear and spatiotemporal patterns of fire occurrence, and they may not generalize well to new situations or regions [2].

Statistical modeling often leads to a numerical index that measures the probability of occurrence of a wildfire given the inputs of the statistical model. Several indices, based on weather variables, have been developed over the past century. Each index is presented in the form of a real-valued function of the explanatory variables.

As examples of statistical models, we cite the Angstrom Index, the Nesterov Index, the Keetch Byram Drought Index, the Baumgartner Index, the Canadian Forest Fire Weather Index, the McArthur Forest Fire Danger Index, the Simple Fire Danger Index, the Fire Danger Index, and the Lebanese Fire Danger Index. We refer the reader to Hamadeh’s thesis [3] for a detailed description and comparison of these indices.

3) *Machine learning models*: These models use data-driven algorithms to learn patterns and rules from training data and apply them to new data. They can handle complex and nonlinear problems and adapt to changing conditions. They can also incorporate various types of data, such as images, videos, texts, and sensors. However, they may suffer from overfitting, lack of interpretability, and high computational costs. We refer the reader to the article by Jain et al. [4] for a large review of the literature on the use of machine learning in the prediction of forest fires.
4) *comparison between wildfire forecasting approaches*: These approaches have different advantages and disadvantages depending on the objectives, data availability, and computational resources of the forest fire prediction problem. Table 1 provides an overall comparison between wildfire forecasting approaches. It is not exhaustive or definitive, but it gives an overview of the main differences between the methods. A more detailed comparison between these approaches would require a systematic review of the literature and a rigorous evaluation of their performance on common datasets and metrics.

**Table 1:** Overall comparison between wildfire forecasting approaches.

Method	Accuracy	Complexity	Data requirement	Advantage	Disadvantage	Additional criteria
Physics-based models	High	High	High	Realistic and detailed simulations	Computationally intensive and complex	Can model fire spread and behavior
Statistical models	Moderate	Low	Moderate	Probabilistic and interpretable estimates	May not capture nonlinear and dynamic phenomena	Can identify fire risk factors
Machine learning models	High	Moderate	High	Flexible and adaptive to various problems and data types	May suffer from overfitting, lack of interpretability, and high computational costs	Can handle complex and nonlinear problems
5) *Limitations of wildfires prediction methods*: We give here, some of the challenges or limitations of forest fire predictors, encountered in the literature [5,6].
  • Data availability and quality: Forest fire prediction requires a large amount of data from various sources such as weather, vegetation, topography, human activities, etc. However, some of these data may be incomplete, inaccurate, outdated, or inconsistent, which can affect the reliability and validity of the prediction models.
  • Model complexity and uncertainty: Forest fire prediction involves many factors and processes that are nonlinear, dynamic, and stochastic. Therefore, the prediction models need to account for the complexity and uncertainty of the fire behavior and spread, as well as the interactions and feedbacks among different factors and scales. However, some of these aspects may be difficult to measure, model, or validate.
  • Model generalization and adaptation: Forest fire prediction needs to be applicable to different regions, scenarios, and conditions. However, some of the prediction models may be based on specific assumptions or parameters that may not hold true for other situations or environments. Therefore, the prediction models need to be tested, calibrated, and updated regularly to ensure their accuracy and robustness.

### 1.2 Fire weather index

The dataset that we will use in this study is composed of the variables used in the calculation of Fire weather index (FWI). Thus, this paragraph serves both to describe the FWI index and to define the variables that compose it.

FWI is one of the world’s most widely used fire weather indices for measuring wildfire risk. This index is based on a Canadian empirical model developed and used in Canada since 1971 [7]. This is calculated from five components that take into account the effects of fuel water content and wind on the behavior of fires:

• the **component 1**, called Fine Fuel Moisture Code and denoted FFMC, measures the moisture content of litter and fine fuels. This index reflects the ease of ignition of the fine fuel,
• the **component 2**, called Duff Humidity Code and denoted DHC, measures the moisture content of organic layers of moderate thickness. This index reflects fuel consumption in moderate humus layers as well as medium-sized woody materials,
• the **component 3**, called Drought Code and denoted DC, measures the moisture content of deep, compact organic layers. This index reflects the effect of seasonal drought on the slow burning of these layers,
• the **component 4**, called Initial Spread Index and denoted ISI, measures the speed at which the wildfire spreads, the component 5, called Buildup Index and denoted BUI, measures the amount of fuel available for combustion.

The first three components are fuel moisture indices, and the other two are fire behavior indices. The calculation of these variables is based on daily measurements of meteorological features such as wind speed, temperature, relative humidity, and precipitation [7]. Apart from Canada, FWI is calculated by the meteorological services of several countries, including China, Chile, Fiji, Indonesia, Malaysia, Mexico City, New Zealand, Portugal, South Africa, Spain, Sweden, Thailand, United Kingdom, and Argentina [3].

### 1.3 Forest fires in Algeria

Among the countries of the Mediterranean basin, Algeria is one of the countries where the problem of forest fires, little known to the scientific community, is acute due to its devastating impact [8]. Compared to countries in North Africa and the Middle East, Algeria is the most affected by forest fires, with a record in 2020 of 69% of the total area burned in these regions. Indeed, if the burned areas are relatively modest compared to other countries around the Mediterranean such as Portugal, Spain, Greece, and Italy, the scarcity of forests and the threat of desertification mean that these wildfires have a disastrous impact on Algeria. Despite the vastness of the Algerian territory, the natural forest occupies only 1439 million ha, with an increase of 0:13% between 2010 and 2020, i.e., an annual area of 1900 ha [9]. The central coastal region of Algeria is characterized by the abundance of significant forest cover [10,11], which presents a very favorable environment for the appearance and spread of fires [[12], [13], [14]]. Indeed, according to the General Directorate of Forests (2018), the central regions of Algeria have 446 936 ha of forest area, of which 10% (44 300 ha) are degraded by wildfires between 2008 and 2017 [15].

### 1.4 Motivation for this study

The southern shore of the Mediterranean basin (the Maghreb including Algeria, Morocco, and Tunisia) has been much less considered than the northern shore, which includes important and known fire zones (Portugal, Spain, Greece, and Italy). It is therefore important to develop wildfire forecasting models for the southern shore of the Mediterranean basin, which may differ from those for the northern shore.

The lack of datasets has limited the methods used to predict wildfires in Algeria to the mapping risk areas, which are updated annually. This study is the result of the availability of a recent dataset relating the history of forest fires in the cities of Bejaia (located in the northeast) and Sidi Bel-Abbes (located in the northwest) during the year 2012. This dataset is the only one officially released as of May 2022. We do not yet have datasets for years before or after 2012. We do not yet have datasets for years before or after 2012. We aim through this work to encourage the Conservation des Forets d’Alger (CFA) to regularly publish this type of database in order to be able to develop more relevant forest fire forecasting models. The preparation of a dataset like the one considered in this study does not require costly logistics. In addition, it allows us to predict with great accuracy the occurrence of a forest fire.

**Fig. 1:**
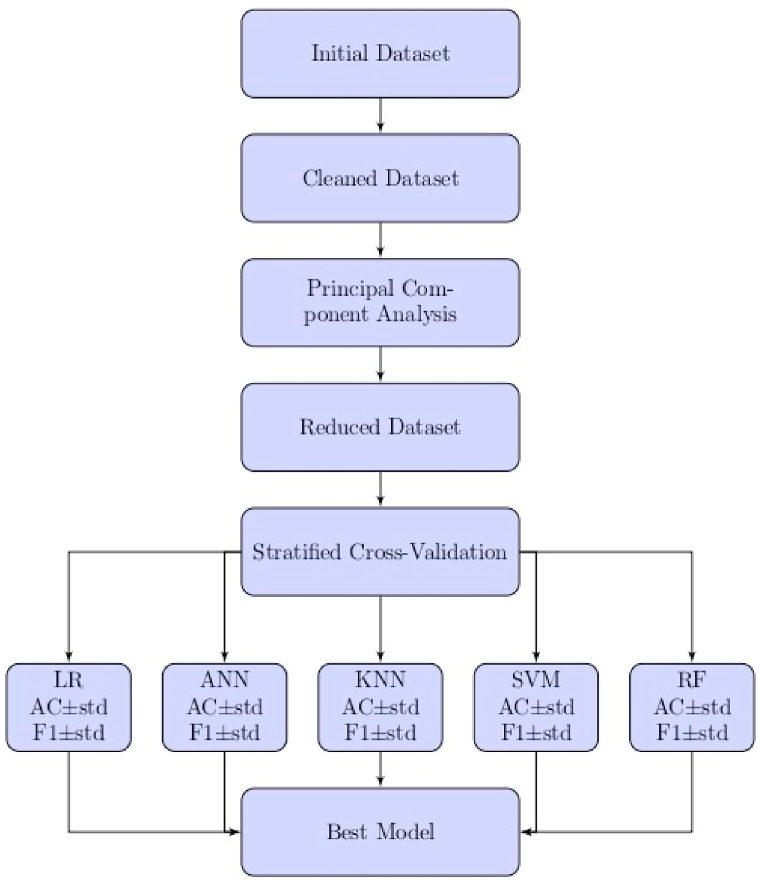
Steps in building a forest fire prediction model, where AC denotes the accuracy, F1 the F1-score, and std the standard deviation.

We propose to use machine learning and data science technique to build a wildfire prediction model, leveraging the information in this dataset. The flowchart presented in Fig. 1 summarizes the approach we follow in this study.

The rest of the manuscript is organized as follows. We reserve Section 2 for the description of the dataset we use in this study. Section 3 provides an overview of the learning models we use to carry out this study. Section 4.1 presents the tools we use to compare the performance of the prediction models. Finally, we present and comment on the results of the experimental study in Section 5.

## 2 Dataset

**Fig. 2:**
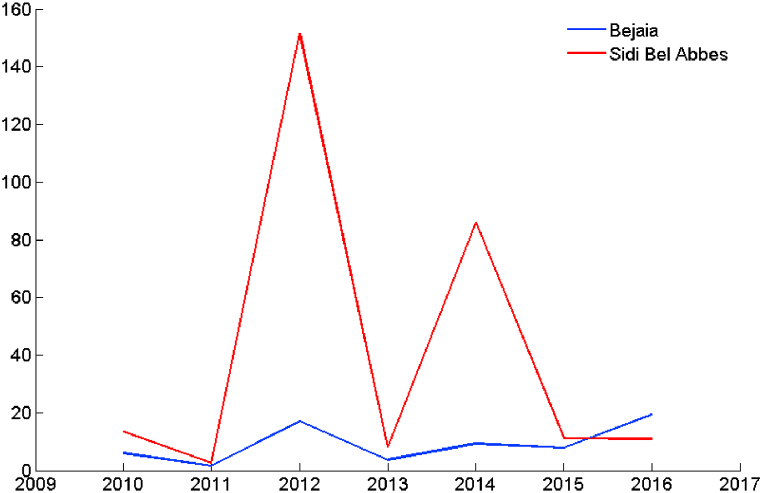
Average area burned in ha per wildfire in the regions of Bejaia and Sidi Bel-Abbes, between 2010 and 2016.

Table 2, Table 3 present the occurrences of forest fires in Bejaia and Sidi Bel-Abbes regions, as well as the area of damage caused by these fires during the years 2010–2016 [16]. Fig. 2 shows the average area burned in ha per wildfire in the regions of Bejaia and Sidi Bel-Abbes, during the years 2010–2016. In view of this figure, these two regions experienced devastating wildfires during the same year 2012. We therefore propose to develop in this study wildfire prediction models based on data relating to the year 2012.

**Table 2:** Number of wildfires and areas burned in the city of bejaia during 2012.

Year	2010	2011	2012	2013	2014	2015	2016
Area (ha)	1967	46	6865	382	3300	544	3946
Occurrence	328	30	399	104	353	70	202

**Table 3:** Number of wildfires and areas burned in the city of sidi bel-abbes during 2012.

Year	2010	2011	2012	2013	2014	2015	2016
Area (ha)	617	102	11 820	1003	15 841	3664	3397
Occurrence	46	40	78	124	184	331	310

The dataset [17] used in this study has been available online since May 2, 2022. The dataset has 10 input quantitative features (Temp, …,FWI) and 1 target feature as shown in Table 4. The output feature is recorded 1 for fire and 0 for no fire. The dataset [17] includes 244 instances evenly distributed across the two regions. The data collection period is from June 1, 2012, to September 30, 2012. Table 4 provides a brief description of the variables involved in this dataset.

**Table 4:** Details of the used dataset.

No	Attribute	Description
1	Temp	Temperature at 12 noon in degrees Celsius
2	RH	Relative Humidity in %
3	Ws	Wind speed in *km/h*
4	Rain	24 h of accumulated precipitation in mm from noon to noon
5	FFMC	Fine Fuel Moisture Code
6	DMC	Duff Moisture Code
7	DC	Drought Code
8	ISI	Initial Spread Index
9	BUI	Buildup Index
10	FWI	Fire Weather Index
11	**Target**	{fire: 1 | no fire: 0}

### 2.1 Wildfire forecast based on the FWI index

**Fig. 3:**
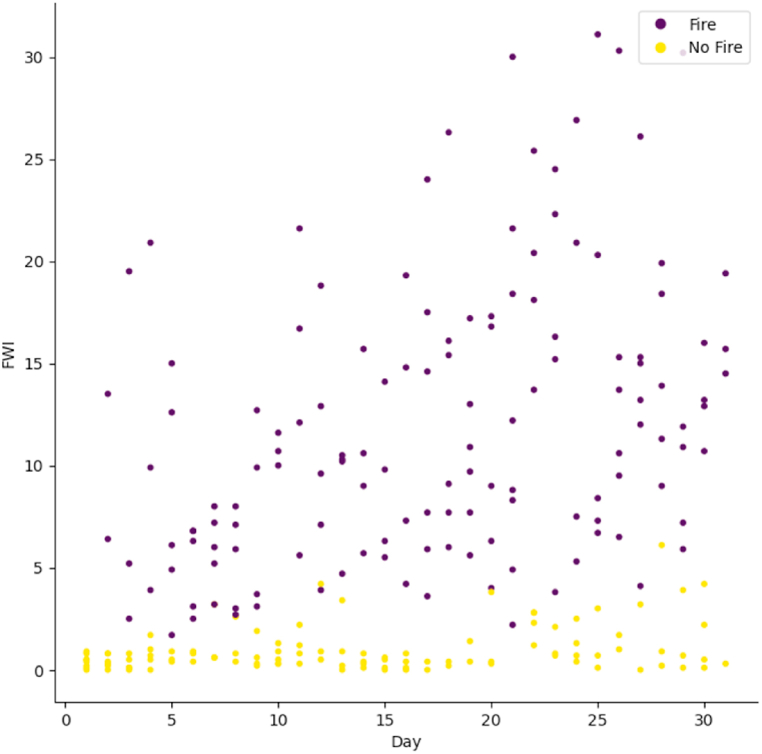
FWI for each day from June 1 to September 30, 2012.

Fig. 3 presents the value of the FWI index for the period from June 1 to September 30, 2012. As can be seen, the higher the FWI, the higher the probability of occurrence of a forest fire. However, it is difficult to determine the threshold which separates the fire and non-fire classes, and which minimizes the number of misclassified occurrences. This is one more reason that prompts us to explore other wildfire forecasting models.

## 3 Methodology

As the dataset contains only 244 observations and the number of parameters of each classifier increase with the number of variables in the dataset, then reducing the number of columns in the dataset leads decreases the complexity of the classifier. Principal component analysis (PCA) is one of the techniques for reducing the number of variables in a quantitative dataset. Thus, in this study, we will use five machine learning classifiers to predict wildfires, and we will apply PCA to reduce the number of parameters of each classifier.

### 3.1 Principal component analysis

PCA is one of the tools of descriptive statistics that can be applied to a dataset involving many individuals and quantitative variables. Concretely, PCA makes it possible to extract the relevant information contained in the dataset and to synthesize it in the form of principal components, new axes to describe the dataset [18]. The principal components are the eigenvectors of the covariance matrix of the data, sorted in descending order of corresponding eigenvalue. The number of principal components to retain depends on the proportion of the total variance explained by these components requested by the user. Thus, the projection of the individuals onto the vector space spanned by the first k principal components constitutes the least distorted representation of the dataset onto a vector space of dimension k. It is often useful to perform PCA on the dataset before building a classification model in order to reduce the number of explanatory variables, and therefore reduce the computational cost of the classification model.

### 3.2 Random forest

Random forest (RF) is a machine learning algorithm, proposed by Leo Breiman in 2001 [19]. This algorithm is quite intuitive to understand, quick to train, and produces generalizable results. The only downside, the random forest is a black box that gives results that are not very explanatory. A random forest is composed of a set of independent decision trees. Each tree has a partial vision of the problem thanks to a double random sampling:

1) a random sampling with replacement, on the observations (the rows of the dataset). This process is called tree bagging,
2) a random sampling, on the variables (the columns of the dataset). This process is called feature sampling.

The prediction made by the random forest for any new input data is nothing but the average (or the vote, in the case of a classification problem) of all these independent decision trees.

In other words, rather than having one complex estimator that can do it all, random forest uses multiple simple estimators (of lower individual quality). Each estimator has a fragmented view of the problem. Then, all these estimators are assembled to obtain the global vision of the problem. It is the assembly of all these estimators that makes the prediction extremely efficient.

1) ***Tree bagging***: Bagging stands for “bootstrap aggregation”. It is a process of random sampling on the observations carried out in three stages:
  a. Construction of m decision trees by randomly sampling m samples of observations,
  b. Training each decision tree,
  c. To make a prediction on new data, one must apply each of the m trees and take the majority among the m predictions.
2) ***Feature sampling***: It is a process of sampling randomly on the variables. By default, we sample $\left\lceil \sqrt{p}\right\rceil$ variables for a problem with p total variables, where ⌈⋅⌉ is the ceil symbol. For a set of randomly sampled variables, a decision tree is created after learning based on the set of randomly sampled observations. This process makes it possible to reduce the correlation between the trees, which could disturb the quality of the results.

### 3.3 Support vector machine

The Support Vector Machine (SVM) is a supervised learning algorithm that aims to find, in a higher-dimensional space, the best hyperplane that divides a data set into two homogeneous subsets [7]. The technique for determining this hyperplane differs from that used by logistic regression. Indeed, if there are several separating planes, logistic regression would propose one, but SVM would determine the best one in the sense that the distance between this hyperplane and the closest points of this hyperplane is maximum. For a given separating hyperplane H, the distance between H and the training points closest to H is called the margin and will be denoted ε. Let $x={\left(x_{1},.\;.\;.,x_{m}\right)}^{t}\in R^{m}$, $w={\left(w_{1},.\;.\;.,w_{m}\right)}^{t}\in R^{m}$, and $w_{0}\in R$, then the Cartesian equation of a separating hyperplane is written as,

$$w^{t}x+w_{0}=0\text{.}$$

We easily prove that,

$$\varepsilon =\frac{2}{∥w∥}\text{,}$$

and that **w** is the unique solution of the following quadratic optimization problem under linear constraints,

$$\min_{w}∥w{∥}^{2}such\;that\;y_{i}\left(w^{t}x+w_{0}\right)\geq 1,i=1,\ldots ,n\text{.}$$

The closest training points to the separating hyperplane are called support vectors. It is proven that the hyperplane given by an SVM depends only on these support vectors.

SVM is particularly effective when the training data size is small. As the dataset contains only 244 observations, SVM will be a priori, a privileged candidate compared to the other classifiers.

### 3.4 K-nearest neighbors

*K*-nearest neighbors (*K*-NN) is a supervised learning algorithm used in both classification and regression problems [20]. It belongs to the class of nonparametric supervised learning methods. Thus, the learning phase is simply reduced to the separation of the dataset according to the modalities of the target variable. To predict the class of a new observation **z**, the *K*-NN algorithm:

a. Calculates, for each observation **x**^*i*^ of the training dataset, the distance between **z** and **x**^*i*^,
b. Retains from the training dataset the closest *K* observations to **z**,
c. Determines the mode of the *K* observations retained and places **z** in the class of the mode.

For this algorithm, the choices of the number *K* and the distance function have a direct impact on the result.

### 3.5 Logistic regression

Logistic regression consists of analyzing the relationship between a binary response variable *Y* and exclusively binary or continuous explanatory variables $X={\left(X_{1},\ldots ,X_{m}\right)}^{t}$, where $x^{t}$ is the transpose of x [21]. The variable Y separates the possible values of X into two disjoint subsets $X_{0}$ and $X_{1}$. The logistic regression is based on the assumption that $X_{0}$ and $X_{1}$ are separated by an affine hyperplane $H\subset R^{m}$. The Cartesian equation of H is defined from m+1 real parameters $w={\left(w_{0},.\;.\;.,w_{m}\right)}^{t}$, such that for all $x={\left(x_{1},.\;.\;.,x_{m}\right)}^{t}\in$ $R^{m}$,

$$x\in H⟺w_{0}+w_{1}x_{1}+\ldots +w_{m}x_{m}=0\text{.}$$

By separating *X*(Ω) by an affine hyperplane, we can assume that:

1. $X_{1}=\left\{x\in R^{m}|w_{0}+w_{1}x_{1}+\ldots +w_{m}x_{m}\;>0\right\}$, corresponds to the class for which Y=1,
2. $X_{0}=\left\{x\in R^{m}|w_{0}+w_{1}x_{1}+\ldots +w_{m}x_{m}\leq 0\right\}$, corresponds to the class for which Y=0.

For a given observation x∈X(Ω), the random variable [Y∣X=x] follows Bernoulli distribution with parameter $S_{W}\left(x\right)$, i.e., $\left[Y|X=x\right]⤳B\left(S_{W}\left(x\right)\right)$, and

$$P\left(Y=1|X=x\right)=S_{W}\left(x\right)\text{,}$$

where P(A) designates the probability of the event A, and $S_{W}\left(\cdot \right)$ is a mapping from $R^{m}$ to [0,1] called score function [22]. Given a threshold θ∈]0,1[, we decide that:

$$\left\{ \begin{array}{ll} x\in X_{1} & if\;S_{W}\left(x\right)\geq \theta \\ x\in X_{0} & if\;S_{W}\left(x\right)<\theta \end{array}\right.$$

The natural and most used value of the threshold is θ=0.5, but nothing prevents taking θ>0.5 if we want the classification to be stricter. There are several candidates for the choice of the function $S_{W}\left(\cdot \right)$. For the logistic model we consider the function,

$$S_{W}\left(x\right)=\sigma \left(w_{0}+w_{1}x_{1}+\ldots +w_{m}x_{m}\right)\text{,}$$

where σ(⋅) is the sigmoid function.

### 3.6 Multilayer perceptron

An Artificial Neural Network (ANN) is an architecture of a large number of interconnected elements called neurons. These neurons process the received input to give the desired output. Nodes or neurons are linked by inputs, connection weights and activation functions. The main characteristic of a neural network is its ability to learn. Neural networks train with known examples. Once the network is trained, it can be used to solve the unknown values of the problem. The neural network learns through various learning patterns classified as supervised or unsupervised learning. In supervised learning algorithms, the target values are known to the network. It tries to reduce the error between the desired output (target) and the actual output for optimal performance. In unsupervised learning algorithms, the target values are unknown, and the network learns by itself from the data, by detecting hidden patterns in the data or by clustering data according to their similarities or differences.

An ANN [23] consists of 3 parts, namely the input layer, hidden layer, and output layer. For an ANN, there is a single input layer, a single output layer, and zero or more hidden layers. Based on this structure, the ANN is classified into single-layer or multi-layer networks respectively.

***1) ANN terminology*:** Here we give a brief description of the parameters and hyper-parameters needed to build any ANN.
a) **Weight:** For an ANN, each neuron is connected to other neurons via connection links. Each link has a weight that contains information about the neuron’s input signal. The weights and the input signal are used to obtain an output. Weights can be represented in a matrix form also called connection matrix. Each neuron is connected to all other neurons in the next layer via connection weights. Therefore, if there are r nodes and each node has c weights, then the weight matrix W is an (*r,c*)-matrix having the following form:

$$W=\left(\begin{array}{ccc} w_{1,1} & \cdots & w_{1,c}\\ \vdots & \ddots & \vdots \\ w_{r,1} & \cdots & w_{r,c} \end{array}\right)$$

where $w_{i,j}$ represents the weight vector from the *i*-th processing element (neuron) to the *j*-th processing element of the next layer, and $w^{i}={\left(w_{i,1},\ldots ,w_{i,r}\right)}^{t}$ represents the weight vector from node *i*.

b) **Bias:** The bias is added to the network by adding an input element *x*_0_ = 1 into the input vector. The bias also has a weight denoted *w*_0_. Bias plays an important role in calculating the output of the neuron. The bias can be positive or negative. A positive bias increases the net weight of the inputs while a negative bias reduces the net inputs.
c) **Threshold:** A threshold value is used in the activation function σ(⋅). The net input is compared to the threshold to get the output:

$$output=\left\{ \begin{array}{ll} 1 & if\;\sigma \left(net\;input\right)\geq threshold\\ 0 & if\;\sigma \left(net\;input\right)<threshold \end{array}\right.\text{.}$$

d) **Learning rate:** It is denoted and varies from 0 to 1. It is used for weight adjustment during the ANN learning process.
e) **Epoch:** We define an epoch as the number of times *K*, that the training dataset passes through the machine learning algorithm. A pass is equivalent to a round trip. The number of epochs can reach several thousand, because the procedure repeats itself indefinitely until the error rate of the model is sufficiently reduced.
f) **Batch:** The learning dataset is usually broken down into batches of the same size p, especially when the dataset is big. Each iteration of a batch neural network algorithm includes calculating the cost function, modifying and back-propagation of all weighting factors. Hence, an iteration involves processing a batch while an epoch means processing all the data in the dataset. The batch size and the number of epochs are hyper-parameters of the learning algorithm and must be given to the algorithm. These parameters should be configured by testing many values to determine which ones are optimal.
g) **Cost function:** Any supervised learning model tends to match output values (or predictions) to input data through learning. This learning results from an optimization algorithm, aiming to obtain optimal weights for each neuron. This optimization is applied to a function, which measures the deviation (or distance) between the target’s prediction and its actual value, called the loss function. Several cost functions have been proposed in the literature, for more details see, for example, the article [24]. For the construction of our ANN, we consider the categorical cross-entropy as the loss function [25].
h) **Optimizer:** The parameter of the machine learning model is the argument that minimizes the loss function. Several machine learning optimizers have been proposed in the literature, for more details see, for example, the article [26]. For the construction of our ANN, we use the Mini-Batch Stochastic Gradient Descent (MBSGD) to perform loss function minimization. We briefly describe how this algorithm is implemented. Let

$$E\left(w|X\right)=\frac{1}{n}\sum_{i=1}^{n}E\left(w|x^{i}\right)\text{,}$$

be the loss function of the model, where $H=\left\{x^{1},\ldots ,x^{n}\right\}$ is the training dataset from which we remove the target variable.

**Gradient Descent (GD):** The parameter w is updated according to the following iterative scheme:

$$w^{\left(k+1\right)}=w^{\left(k\right)}-\alpha \nabla E\left(w^{\left(k\right)}|X\right)\text{,}$$

where $w^{\left(k\right)}$ represents the value of parameter w at iteration *k*, $\nabla E\left(w^{\left(k\right)}|X\right)$ is the gradient of *E* with respect to **w**, and α is the learning rate.

**Stochastic gradient descent (SGD):** It exploits the fact that loss function is an average over independent and identically distributed (i.i.d.) observations to make updates of w much more frequent. At the iteration *k*, we randomly select an observation **x** in X, and we update **w** according to the following scheme:

$$w^{\left(k+1\right)}=w^{\left(k\right)}-\alpha \nabla E\left(w^{\left(k\right)}|x\right)\text{,}$$

Stochastic gradient descent is a more general principle, in which the direction of update is a random variable, whose expectation is the true gradient of interest.

**MBSGD:** It is a variant of SGD, in which the direction of the update is obtained by averaging the loss function over a subset *B* of X called the mini-batch. Let *p* be the size of a mini-batch, then, for each epoch, the training dataset is randomly divided into np mini-batches. The weight vector **w** of the model is updated for each mini-batch. Therefore, along the same epoch, the parameter **w** is updated np times. Using *K* epochs, the model will explore the training dataset *K* times, i.e., a total of Knp mini-batches during the whole training process. So, for a mini-batch B, the parameter w is updated according to the following iterative scheme:

$$w^{\left(k+1\right)}=w^{\left(k\right)}-\alpha \nabla E\left(w^{\left(k\right)}|B\right)\text{,}$$

where,

$$E\left(w|B\right)=\frac{1}{card\left(B\right)}\sum_{x^{i}\in B}E\left(w|x^{i}\right)\text{.}$$

## 4 Evaluation of prediction models

We present in this part the tools that we will use to evaluate the performance of the prediction models.

### 4.1 Metrics

To evaluate a classifier on the test dataset, we consider the commonly used performance indices, namely accuracy, precision, recall, and F1-score [27,28]. Let *n*_*t*_ be the number of instances of the test dataset. Denote by *n*_0,0_, *n*_0,1_, *n*_1,0_, *n*_1,1_ be a partition of *n*_*t*_ such that:

1. *n*_0,0_ is the number of instances of the test dataset whose class is 0 and which are correctly predicted by the prediction model,
2. *n*_0,1_ is the number of instances of the test dataset whose class is 0 and which are falsely predicted by the prediction model,
3. *n*_1,0_ is the number of instances of the test dataset whose class is 1 and which are falsely predicted by the prediction model,
4. *n*_1,1_ is the number of instances of the test dataset whose class is 1 and which are correctly predicted by the prediction model,
5. *n*_0,0_, *n*_0,1_, *n*_1,0_, *n*_1,1=_*n*_*t*_*.*

Using the notations above, we define the performance indices as follows:

1) The **accuracy** of the model is denoted AC and defined as the ratio of the number of correctly predicted instances by the total number of instances, i.e.,

$$AC=\frac{n_{0,0}+n_{1,1}}{n_{t}}\text{.}$$

2) The **precision** of the model is denoted PC and defined as the ratio of correctly predicted wildfires to the total number of predicted wildfires, i.e.,

$$\mathrm{PR}=\frac{n_{1,1}}{n_{0,1}+n_{1,1}}\text{.}$$

3) The **recall** of the model is denoted RE and defined as the ratio of correctly predicted wildfires to the total number of wildfires instances, i.e.,

$$RE=\frac{n_{1,1}}{n_{1,0}+n_{1,1}}\text{.}$$

4) The **F1-score** is defined as the harmonic mean of precision and recall, i.e.,

$$F1-score=\frac{2}{\frac{1}{\mathrm{PR}}+\frac{1}{RE}}$$

The F1-score is a metric that measures the model’s ability to predict wildfires well, both in terms of precision and recall. This score is between 0 and 1, and the closer it is to 1, the more efficient the model is.

### 4.2 Training and test datasets

Usually, we split the dataset into two disjoint subsets. The larger subset, called the training set, is used to learn the classifier, and the smaller one, called the test set, is used to measure the performance of the classifier on unseen data. In this way, the performance indices introduced above are calculated based on the test set. Although this approach is inexpensive in terms of CPU time, it may seem a bit subjective, as it does not take into account the extreme distributions of the dataset ([29], p. 99). To properly evaluate the classification model, it is recommended to consider the following techniques during the validation phase.

1) *C-fold Cross validation:* Too bad, we only used part of the dataset for training and its complementary part to test the model. What if we had by chance created a test set that was really hard or really easy to predict? The performance estimate would be biased. *C*-fold Cross-validation will allow us to use all our data for training and for testing. Thus, *C*-fold cross-validation, splits the dataset into C roughly equal parts. Each of the *C* parts is used alternately as a test set, while the remainder (i.e., the union of the other (*C*−1) parts) is used for training. At the end, each observation served once in a test set and (*C* −1) times in a training set. This way, cross-validation does not violate the non-validation principle on the training set. Finally, we can report the performance of our model by averaging the performance obtained over the *C* folds, in which case we can also report the standard deviation, to quantify the variation in this performance over the *C* folds. For the choice of *C*, the values {3,5,10} are in common use. However, Kohavi [30] recommends using 10-fold cross-validation even when it is computationally possible to use more folds. Therefore, in the experimental part, we will take *C* = 10.
2) *Stratification:* In the case of a classification problem, we generally try to create the C folds so that they follow approximately the same distribution as the complete dataset. We try to avoid that a training set contains only positive examples, and the corresponding test set contains only negative examples, which will negatively affect the performance of the model. This process is called stratified cross-validation (SCV).
3) *Shuffling:* When applying a SCV, it is recommended to split the dataset randomly. For this, we need to set the shuffle option of the SCV to True. Also, if we want to ensure the SCV reproducibility, we need to set the random_state parameter of the SCV to a known value. In the experimental part, we will take random_state = 3.

### 4.3 Impact of variables on model prediction

We can see a supervised learning model as a black box. For a given individual, the values associated with it constitute the inputs of this black box, and the prediction of its class is given by the output of this black box. At the end of the learning phase, we try to find answers to the following questions:

1. How do the inputs affect the predicted class?
2. What are the main inputs that influence the predicted class?

The evaluation metrics introduced in Section 4.1 help us to understand the overall performance of a model. However, we need more details on the impact of different variables on model predictions. There are several techniques that meet this need. In this study, we will opt for the Shapley value (SHAP), which is one of the most widespread model explanation techniques in the literature. Our choice for the SHAP technique is based on the following arguments:

1. **Overall Interpretability:** SHAP values not only show the importance of an explanatory variable but also whether the variable has a positive or negative impact on the prediction.
2. **Local interpretability:** It is possible to calculate the SHAP values for each individual and to know the rate of contribution of each variable to this prediction. This option is not available with the other techniques, which only display aggregated results across the dataset.
3. **Adaptability:** SHAP values can be applied to a wide variety of models, while other techniques are only applied to particular models.

## 5 Experimental study

To conduct the experimental study, we installed Anaconda version 3 which comes with the Python 3+ programming language. To code in Python, we opted for Jupyter Notebook, which is a powerful tool for developing and interactively presenting data science projects.

To build our classifiers, we used popular data science and machine learning libraries. Specifically, we imported:

• the Pandas library to read and manipulate the data saved in the CSV file,
• the Numpy library to convert the data into a format compatible with the inputs of our classification models,
• the Seaborn and Matplotlib libraries to visualize the results,
• the Sklearn library to use the built-in classifiers, and to calculate the k-fold cross-validation performance of the different models,
• the Pickle library to save our models for future use.

All Python scripts are run on a laptop equipped with an Intel dual-core i7-4510U processor, clocked at 2.0 GHz, 8 GB of RAM, a 1 TB hard disk, and Windows operating system 8.1.

### 5.1 Hyper-parameters of the ANN classifier

**Fig. 4:**
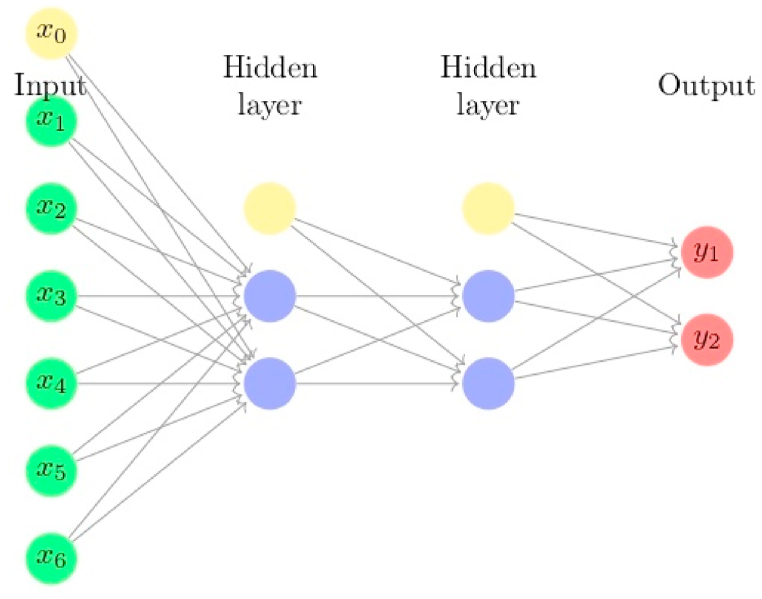
Architecture of ANN with two hidden layers. Each yellow node refers to a bias. This model has 24 parameters, including biases.

We consider the ANN whose architecture is presented in Fig. 4, such that:

• the hidden layers activation function is the softmax,
• the output layer activation function is the sigmoid,
• the input and the hidden layers include a bias neuron,
• the batch size equals 244, and an epoch equals 100. These values are determined experimentally after having tested several combinations of the pair (batch, epoch),
• the optimizer is the stochastic gradient descent. In fact, we tested SGD and ADAM, and retained SGD because it gives better results. Note that when the batch size equals the number of observations, then SGD coincides with GD. Moreover, the results stabilize after 100 epochs, so there is no need to go further.

### 5.2 Optimal value of K for KNN classifier

**Fig. 5:**
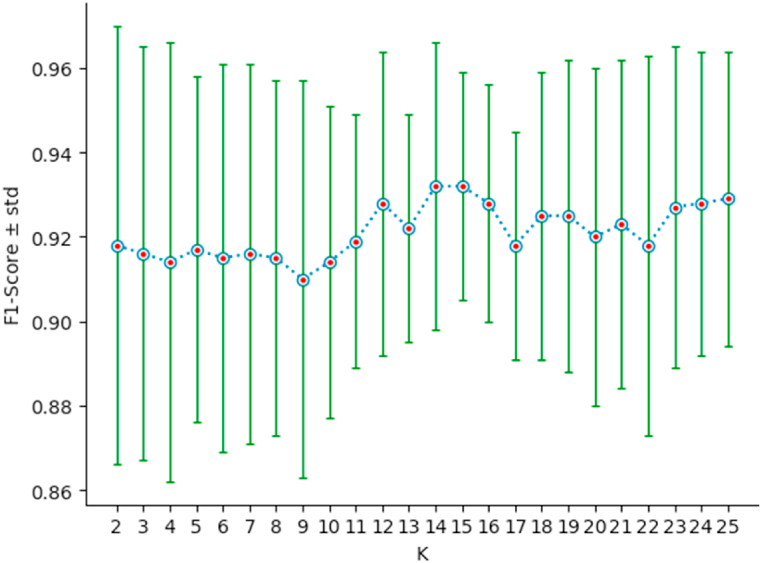
$\bar{F1-score}$ versus *K*, for *K* = 1, …, 25.

To determine the optimal value of *K*, we proceed as follows. For all *K* = 2, …,25, we calculate the mean $\bar{F1-score}$, and the standard deviation std, of the F1−score resulting from the stratified cross-validation of KNN on the dataset. We then retain the value of *K* which maximizes $\bar{F1-score}$ and minimizes std. According to Fig. 5, the optimal value of *K* is 15. Therefore, in the following, the symbol KNN designates the classifier 15-NN.

**Fig. 6:**
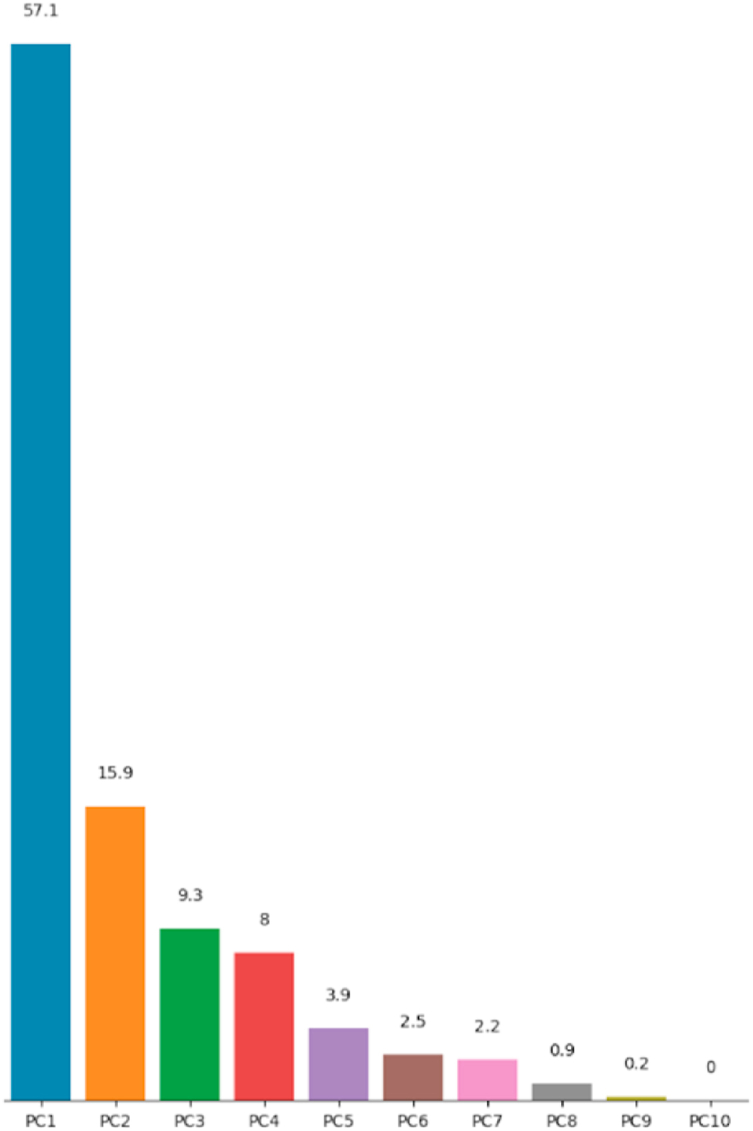
Percentages of variance explained by principal components.

In summary, we use the following notations to refer to classification models:

• LR: logistic regression,
• ANN: artificial neural network model whose architecture is given by Fig. 4,
• KNN: 15-NN,
• SVM: support vector machine,
• RF: random forest.

### 5.3 Principal component analysis outcomes

**Fig. 7:**
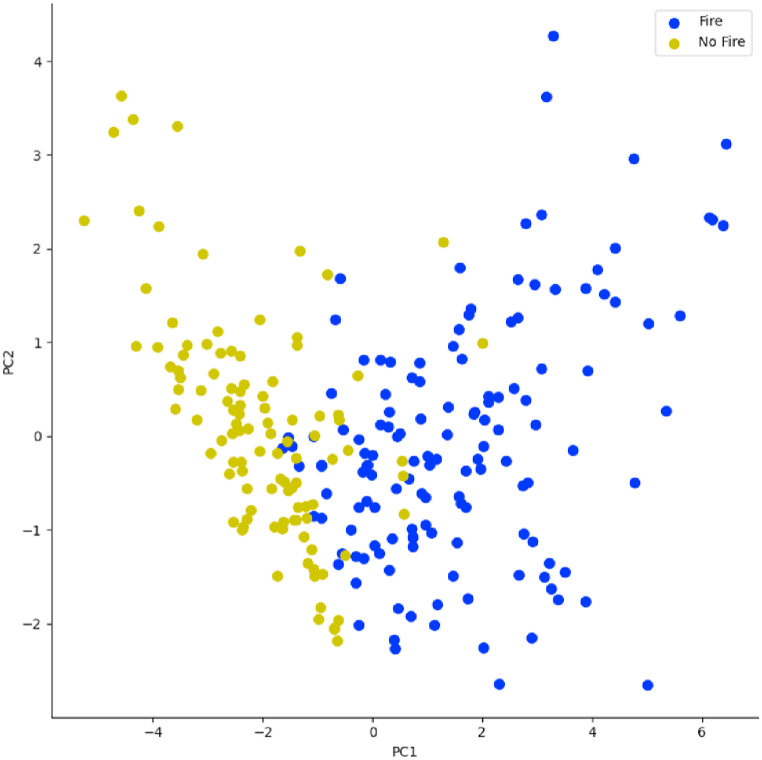
Orthogonal projection of observations in the first principal plane.

**Fig. 8:**
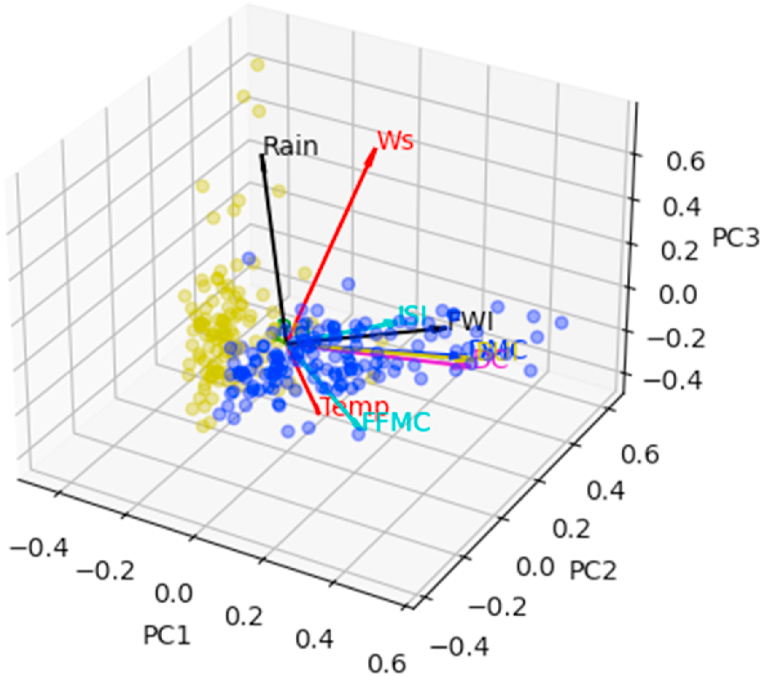
Correlation sphere in the principal space (PC1,PC2,PC3). The blue color refers to the fire class.

Fig. 7 presents the projection of observations on the first principal plane. This figure reveals the existence of two classes separated by a line. These classes are best seen in the first principal space given by Fig. 8, which reinforces the hypothesis that the variables of our dataset are able to separate the observations by a plane of higher dimension.

For all *i* = {1,3,5,7,9}, denote by *C*_*i*_ the correlation circle on the principal plane (PC_i_,PC_i+1_). Inside the circle, each variable is represented by a line segment pointing to the variable name. Well-represented variables in the plane (PC_*i*_, PC_*i*+1_) will have a segment of length close to 1, and their extremity will therefore be close to the circle *C*_*i*_. Thus, it is best to interpret only those variables that are well represented in a principle plan. The angle between two variables gives an indication of their correlation in the principal plane (PC_*i*_, PC_*i*+1_). Thus, an acute angle reveals a positive correlation, a right angle indicates the absence of correlation, and an obtuse angle indicates a negative correlation between the variables. Similarly, the angle between the principal component PC_*i*_ (resp. PC_*i*+1_) and the variable *X*_*k*_, *k* = 1, …,10, is interpreted in the same way as an angle between two variables in this principal plane.

**Fig. 9:**
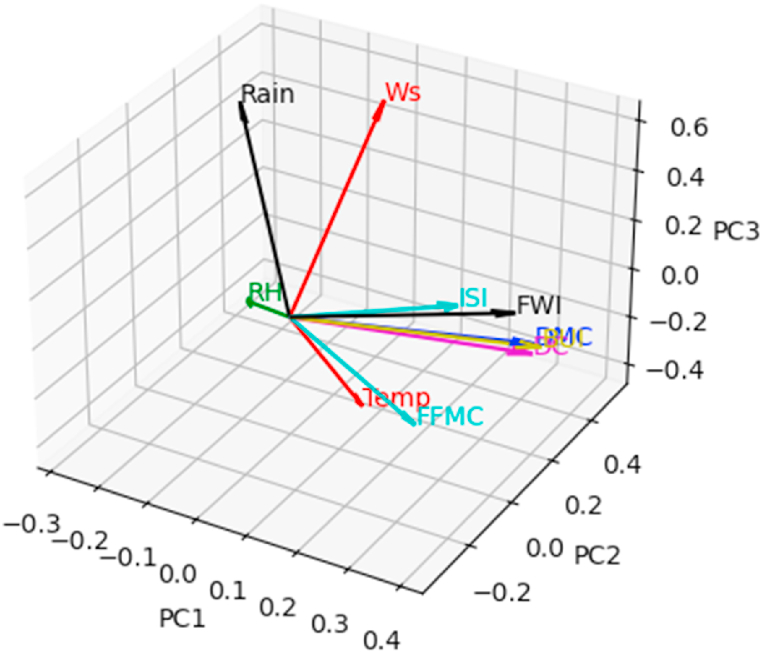
Representation of explanatory variables in the principal space PC1,PC2,PC3.

**Fig. 10:**
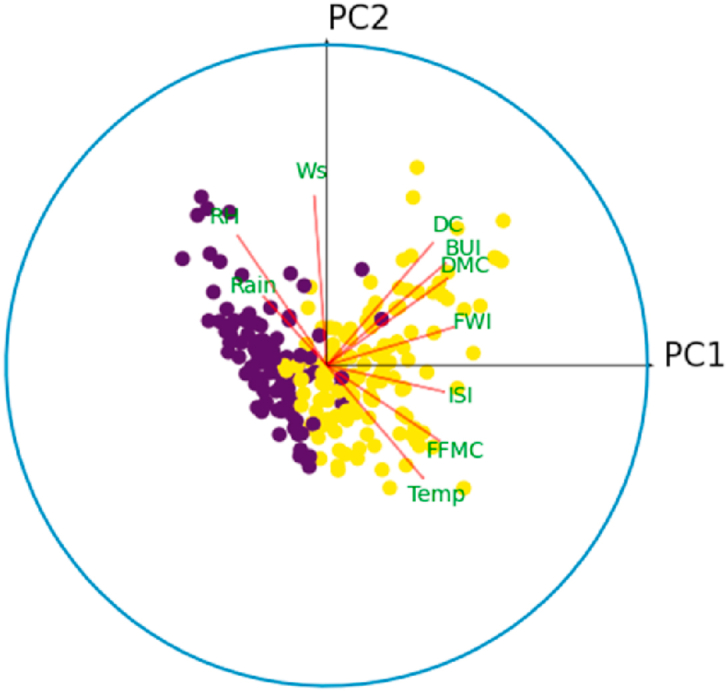
Correlation circle on the principal plane (PC1,PC2). The blue color refers to the fire class.

**Fig. 11:**
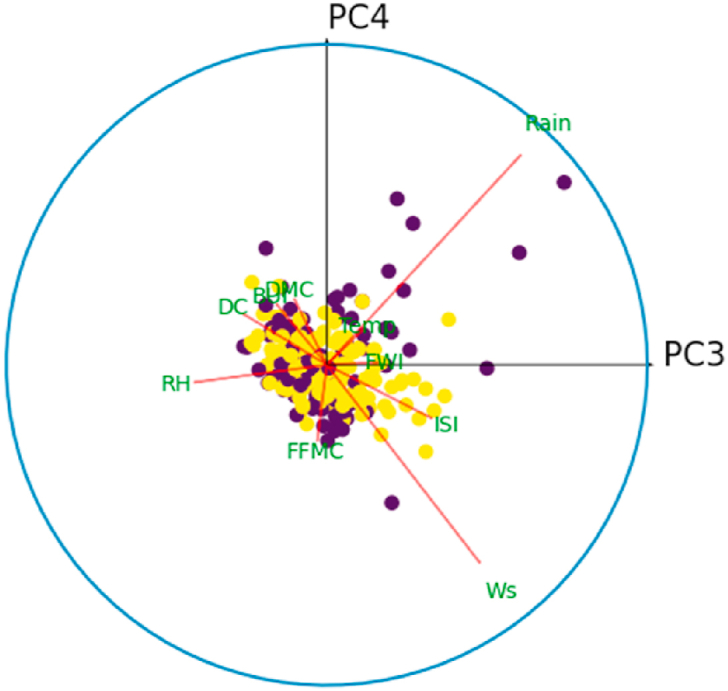
Correlation circle on the principal plane (PC3,PC4). The blue color refers to the fire class.

**Fig. 12:**
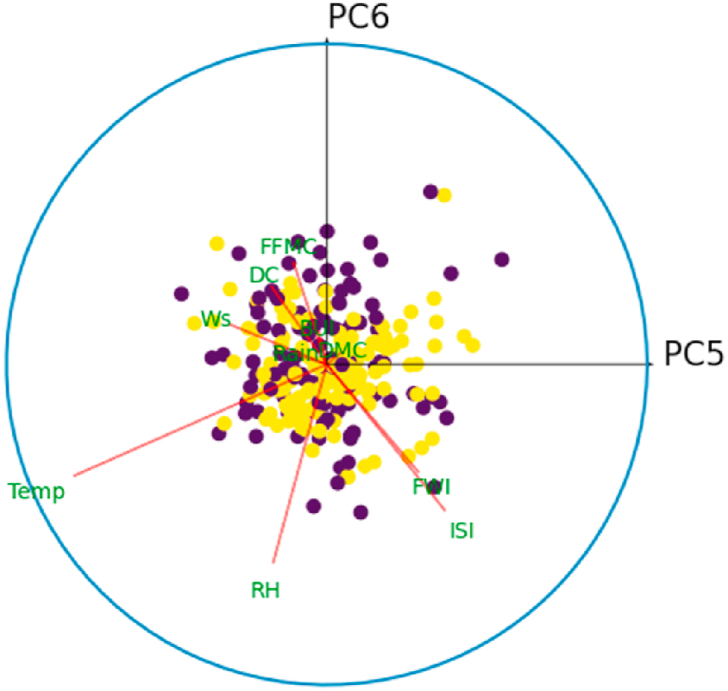
Correlation circle on the principal plane (PC5,PC6). The blue color refers to the fire class.

**Fig. 13:**
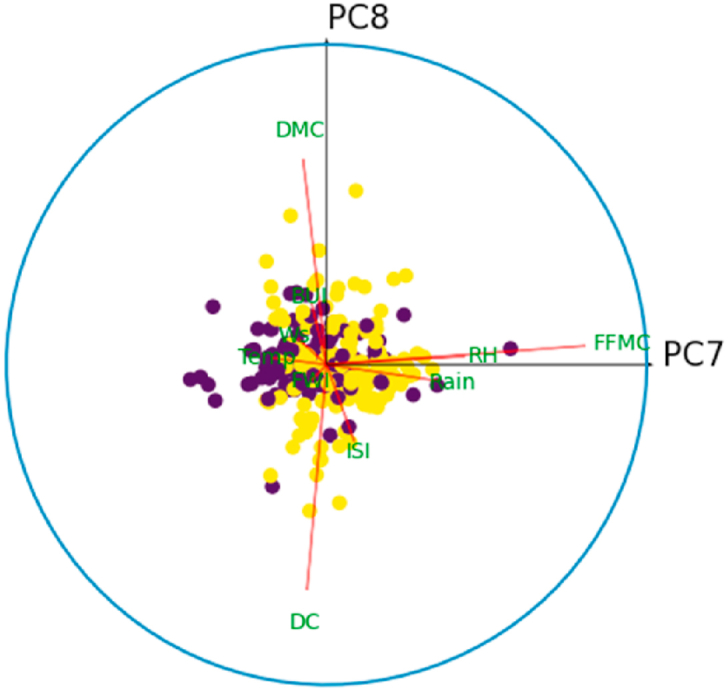
Correlation circle on the principal plane (PC7,PC8). The blue color refers to the fire class.

**Fig. 14:**
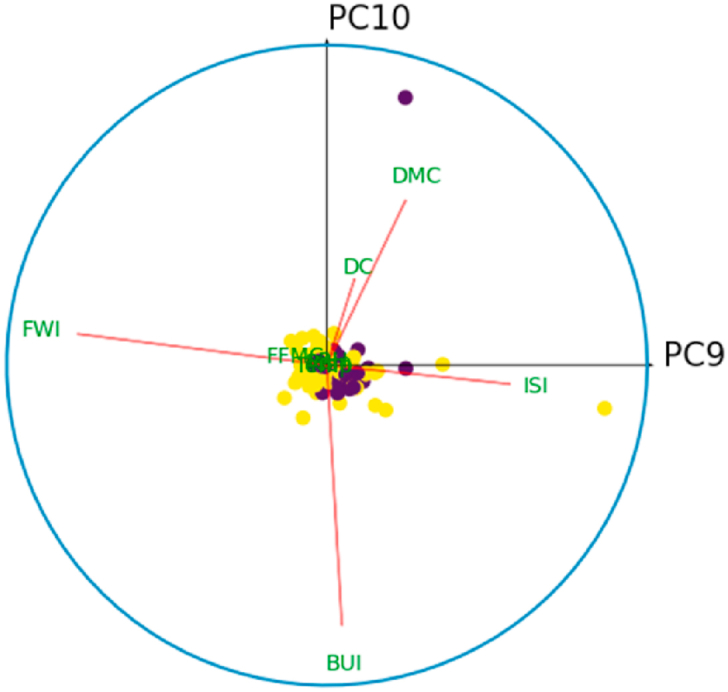
Correlation circle on the principal plane (PC9,PC10). The blue color refers to the fire class.

Fig. 10 shows the correlation circle on the principal plane (PC1,PC2). In this figure, the observations are represented by their orthogonal projections on this plane, while the variables are represented by their correlations [31]. As we can see, the variables are a little far from the circle and therefore poorly represented. This is why we preferred to plot the observations and the variables in the space (PC1,PC2,PC3), to give a more robust interpretation to this graphic illustration. Fig. 8, Fig. 9 show the correlation sphere on the space (PC1,PC2,PC3). As we can see, except for RH, the variables are well represented in these graphics. In view of Fig. 10, Fig. 11, Fig. 12, Fig. 13, Fig. 14, the best representation of the variable RH is given by Fig. 12. It is therefore necessary to retain at least the first six principal components.

Colored bars, presented in Fig. 6, show the percentage of variance explained by each principal component. Thus, PCA reveals that 96.65% of the dataset variance can be represented in a 6-dimensional space. So, we managed to reduce the number of variables from 10 to 6 while retaining nearly 97% of the variance contained in the original dataset. It is therefore necessary and sufficient to retain the first six principal components to reduce the dimension of the dataset without distorting it. The rest of this study will be carried out on the new dataset built from the variables {PC1, …,PC6,Target}.

According to Fig. 14, the FWI index is best represented by the principal component PC9. Although PC9 is excluded from the reduced dataset, the performance of the different models was not affected by this exclusion. This proves that the FWI index alone is insufficient to predict the occurrence of a forest fire.

### 5.4 Performance comparison of different classifiers

**Fig. 15:**
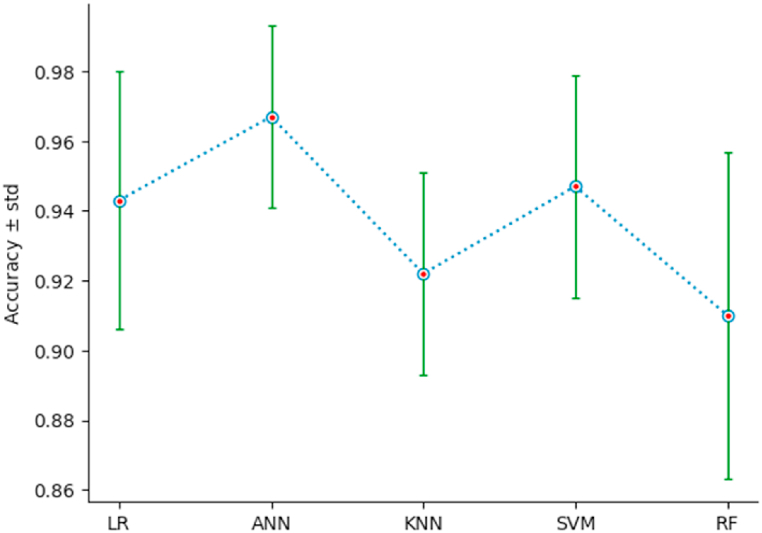
$\bar{AC}\pm std$ for the different classifiers.

**Fig. 16:**
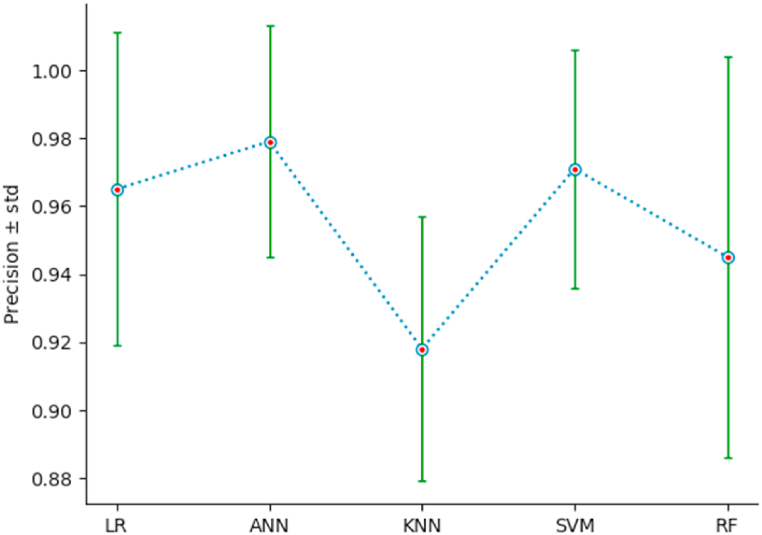
$\bar{\mathrm{PR}}\pm std$ for the different classifiers.

**Fig. 17:**
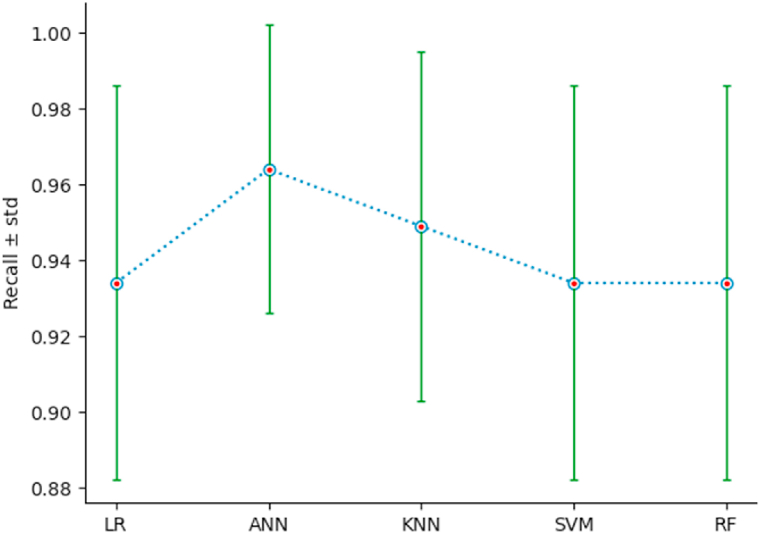
$\bar{RE}\pm std$ for the different classifiers.

**Fig. 18:**
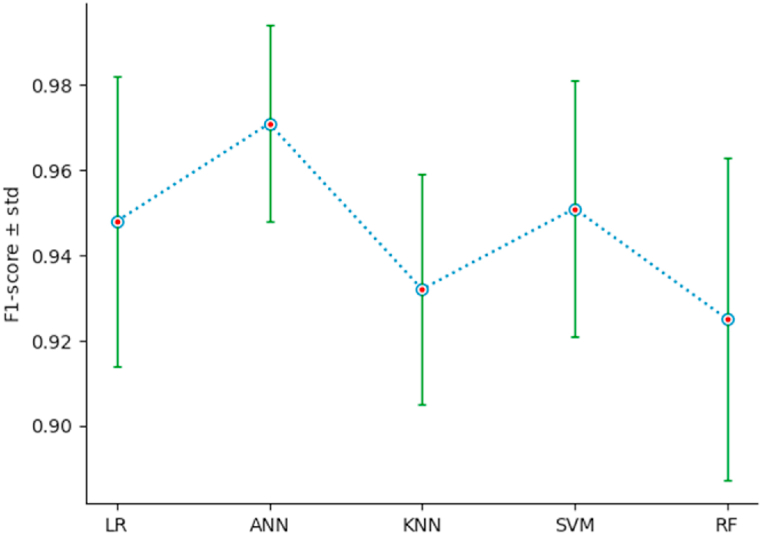
$\bar{F1-score}\pm std$ for the different classifiers.

To measure the performance of the different classification models, we use a 10-fold stratified cross-validation, setting shuffle = True and random_state = 3. The list of performance indices {Accuracy: AC, Precision: PR, Recall: RE, F1-Score} has been defined in section 4.1. For each classifier, and for each index, we calculate the mean and the standard deviation std, of the index resulting from the stratified cross-validation of the classifier on the new dataset {PC1, …,PC6,Target}. The outcomes of this comparison are illustrated by Table 5, and Fig. 15, Fig. 16, Fig. 17, Fig. 18. As we can see, ANN is the classifier that maximizes the mean and minimizes the standard deviation of the four performance indices. For the other classifiers, SVM ranks second, LR third, KNN fourth, and RF last.

**Table 5:** Performances using stratified cross-validation.

Method	AC ± std	PR ± std	RE ± std	F1-score ± std
LR	0*.*943 ± 0*.*037	0*.*965 ± 0*.*046	0*.*934 ± 0*.*052	0*.*948 ± 0*.*034
ANN	0*.*967 ± 0*.*026	0*.*979 ± 0*.*034	0*.*964 ± 0*.*038	0*.*971 ± 0*.*023
*K*-NN	0*.*922 ± 0*.*029	0*.*918 ± 0*.*039	0*.*949 ± 0*.*046	0*.*932 ± 0*.*027
SVM	0*.*947 ± 0*.*032	0*.*971 ± 0*.*035	0*.*934 ± 0*.*052	0*.*951 ± 0*.*030
RF	0*.*910 ± 0*.*047	0*.*945 ± 0*.*059	0*.*934 ± 0*.*052	0*.*925 ± 0*.*038

### 5.5 Analysis by the SHAP technique

The graphic outputs of the SHAP technique are multiple. We report here only the one that shows the feature importance using the summary_plot method. In this plot, the x-axis represents the mean of the absolute SHAP value of each variable, and the variables are ranked vertically according to their influence on the model’s prediction.

**Fig. 19:**
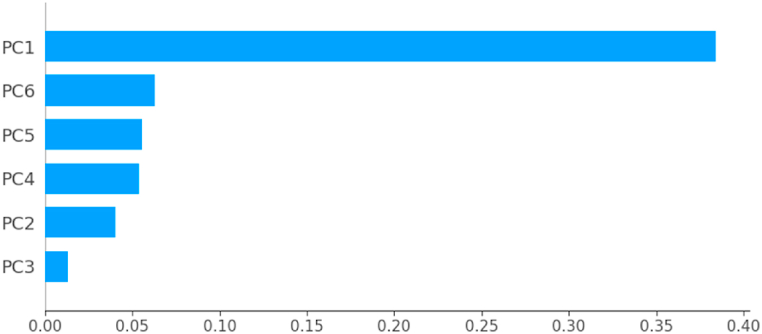
The mean of the absolute SHAP value of the variables PC1, …, PC6 for the classifier LR.

**Fig. 20:**
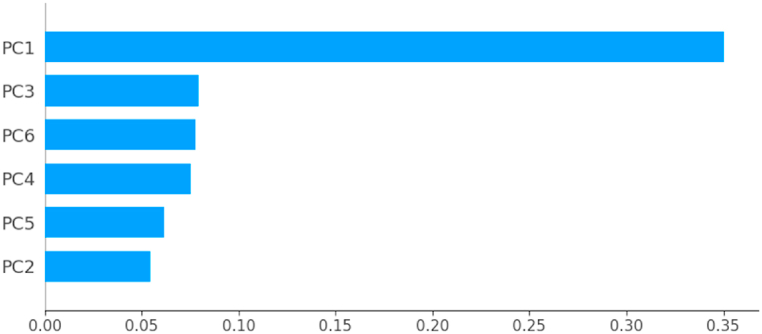
The mean of the absolute SHAP value of the variables PC1, …, PC6 for the classifier ANN.

**Fig. 21:**
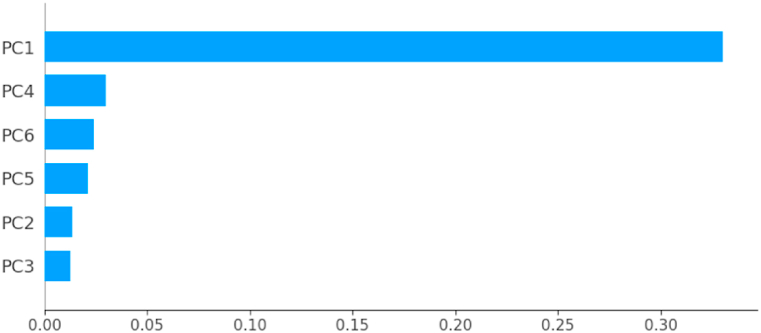
The mean of the absolute SHAP value of the variables PC1, …, PC6 for the classifier K-NN.

**Fig. 22:**
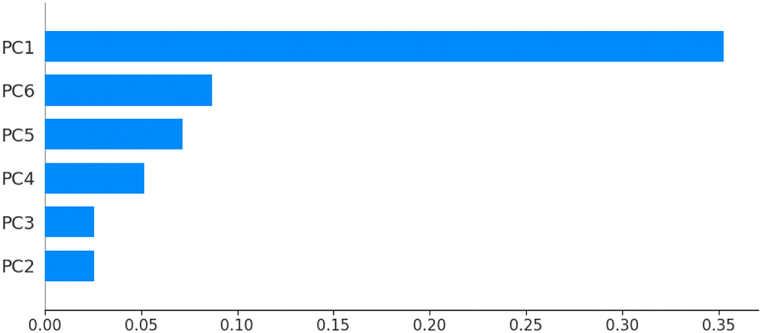
The mean of the absolute SHAP value of the variables PC1, …, PC6 for the classifier SVM.

**Fig. 23:**
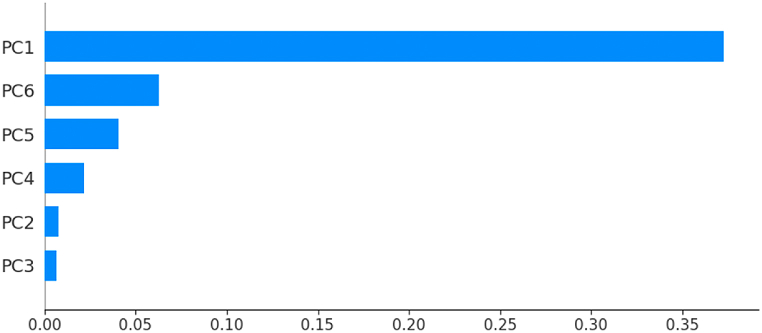
The mean of the absolute SHAP value of the variables PC1, …, PC6 for the classifier RF.

According to Fig. 19, Fig. 20, Fig. 21, Fig. 22, Fig. 23, the principal component PC1 dominates the other components, all models combined. Moreover, the ANN model differs from the other models in that the components (PC2, …, PC6) contribute almost equally to the prediction of fire or non-fire classes.

Table 6 presents the coordinates of each principal component in the basis {Temp,RH,Ws, …,FWI} introduced in Table 4. With the exception of the variable Ws, all the other variables contribute approximately equally to the construction of the first principal component. From Fig. 20, the principal components PC3 and PC6 contribute equally to the prediction of the ANN model. Moreover, the variables, (RH, DC, ISI) contribute to the construction of PC3 and PC6 with almost equal weights. We deduce that the variables (RH, DC, ISI) contribute largely to the predictions of the ANN model.

**Table 6:** Coordinates of the first six principal components.

Variable	PC1	PC2	PC3	PC4	PC5	PC6
Temp	0.299	−0.350	0.110	0.106	−0.786	−0.346
RH	−0.277	0.403	−0.411	−0.054	−0.166	−0.616
Ws	−0.039	0.526	0.475	−0.615	−0.300	0.123
Rain	−0.197	0.214	0.603	0.652	−0.082	0.027
FFMC	0.349	−0.234	−0.029	−0.235	−0.103	0.315
DMC	0.3744	0.2696	−0.100	0.203	0.045	0.0368
DC	0.329	0.380	−0.257	0.157	−0.171	0.241
ISI	0.363	−0.082	0.325	−0.165	0.367	−0.453
BUI	0.370	0.318	−0.155	0.187	−0.024	0.098
FWI	0.394	0.118	0.157	0.007	0.284	−0.3342

## 6 Conclusion

In this study, we developed an ANN with two hidden layers to predict wildfires in the cities of Bejaia and Sidi Bel-Abbes, Algeria. We trained and compared the performance of our classifier with those provided by the LR, KNN, SVM, RF classifiers, using a 10-fold stratified cross-validation. The experiment shows a slight superiority of the ANN classifier compared to the others, in terms of accuracy, precision, and recall. Our classifier achieves an accuracy of 0*.*967 ± 0*.*026, and an F1-score of 0*.*971 ± 0*.*023. Moreover, we have shown, experimentally, that the FWI index, which is used by several countries to predict wildfires, is not sufficient to give reliable predictions in these cities, especially when it falls in the interval [1,4]. Finally, the SHAP technique for interpreting learning models revealed the importance of the variables (RH, DC, ISI) in the predictions of the ANN model.

## 7 Future work

If we had a larger dataset, we could have come up with an ANN with more neurons, or even developed an ANN to predict forest fires for each month. This approach is promising, because already with a small dataset we were able to build a fairly relevant prediction model. The database that we used in this study is the only one published to date in Algeria. In view of the results we have obtained, we hope that the Conservation des Forets^ d’Alger will agree to regularly publish this type of database so that we can develop more relevant forest fire forecasting models.

## Author contribution statement

Abdelhamid Taieb ZAIDI: Conceived and designed the experiments; Performed the experiments; Analyzed and interpreted the data; Contributed reagents, materials, analysis tools or data; Wrote the paper.

## Data availability statement

Data included in article/supp. material/referenced in article.

## Declaration of competing interest

The authors declare the following financial interests/personal relationships which may be considered as potential competing interests:

Abdelhamid ZAIDI reports financial support was provided by Qassim University College of Science.
